# Boost modalities in cervical cancer: dosimetric comparison between intracavitary BT vs. intracavitary + interstitial BT vs. SBRT.

**DOI:** 10.1186/s13014-023-02295-4

**Published:** 2023-06-28

**Authors:** Sofian Benkhaled, Kadiatou Diakité, Nicolas Jullian, Sara Poeta, Christophe Vandekerkhove, Paul Van Houtte, Dirk Van Gestel, Alex De Caluwé

**Affiliations:** 1 Institut Jules Bordet, Department of Radiation-Oncology, Université Libre de Bruxelles, Brussels, Belgium; 2 Department of Radiation Oncology, Lausanne University Hospital and University of Lausanne, UNIL-CHUV, Lausanne, Switzerland; 3 Department of Radiation-Oncology, National Center for Medical Oncology and Radiotherapy Alassane, Abidjan, Ivory Coast; 4 Department of Medical-Physics, Institut Jules Bordet-Université Libre de Bruxelles, Brussels, Belgium

**Keywords:** Cervical Cancer, Brachytherapy, Stereotactic body Radiotherapy, Boost modalities

## Abstract

**Purpose / objective:**

This study compares the dosimetric plans of three distinct boost modalities in cervical cancer (CC): intracavitary (IC) with tandem/ovoids brachytherapy (BT), IC + interstitial (IS) BT, and Stereotactic-Body-Radiotherapy (SBRT). The aim is to determine the dosimetric impact in terms of target coverage and organ at risk (OAR) doses.

**Materials and methods:**

24 consecutive IC + IS BT boost treatment plans were retrospectively identified. For each plan included, two additional plans were created: IC-BT and SBRT. Importantly, no planning target volume (PTV) or planning (organ at) risk volume (PRV) margins were generated, therefore all structures were identical for any boost modality. Two different normalizations were performed: (1) Normalization to the target: prescription of 7.1 Gy to the D90% (defined as the minimum dose covering 90%) of the high-risk clinical target volume (HR-CTV); (2) Normalization to the OARs. HR-CTV coverage and OARs sparing were compared. The equivalent doses in 2 Gy fractions (EQD2) of EBRT and BT for CTV-HR and OARs were calculated using the linear-quadratic model with α/β of 10 (EQD2_10_) and 3 (EQD2_3_), respectively

**Results:**

A total of 72 plans were investigated. In the first normalization, the mean EQD2_3−_D2cc (defined as the minimal dose of the 2 cc) of OAR was significantly higher in the IC-BT plans, and the bladder D2cc hard constraint could not be reached. IC + IS BT leads to a 1 Gy mean absolute decrease of bladder EQD2_3_-D2cc (relative dose: -19%), allowing to reach the hard constraint. SBRT (without PTV) delivers the lowest EQD2_3_-D2cc to the OAR. In the second normalization, IC-BT provides a significantly lower dose to the EQD2_10_-D90% (6.62 Gy) and cannot achieve the coverage goal. SBRT (without PTV) yields the highest dose to the D90% of HR-CTV and a significantly lower EQD2_10_-D50% and D30%.

**Conclusion:**

The key dosimetric benefit of BT over SBRT without PTV is a significantly higher D50% and D30% in the HR-CTV, which increases the local and conformal dose to the target. IC + IS BT vs. IC-BT provides significantly better target coverage and a lower dose to the OARs, making it the preferred boost modality in CC.

## Introduction

Cervical cancer is the fourth most common disease in women and the fourth cause of cancer mortality [1]. Pelvic external beam radiotherapy (EBRT) with concomitant chemotherapy (CCRT) and brachytherapy (BT) is the current standard of treatment for locally advanced cervical cancers (LACC) [2–5]. In cervical cancer (CC), the shift from 2D to 3D and BT treatment planning reduced toxicity and increased local control (LC) and overall survival (OS) [6–8]. As a result, a shift from an empirical prescription (Point A) to a 3D target (high-risk CTV or HR-CTV) was possible [9]. In the EMBRACE-I study, the 5-year LC with MRI-based image-guided adaptive BT (IGABT) in the early (IB-IIB) and advanced (IIIA-IVB) stages, was reported as 91–98% and 100–89%, respectively [10]. Moreover, the five-year treatment-related morbidity (≥ grade 3) rate was around 3.28.5% [10].

When it is not possible to perform BT (patient or treatment facility reasons), EBRT or Stereotactic body radiotherapy (SBRT) cervix boost (16–36 Gy/1.8-6 Gy per fraction) has been used (instead of BT) in up to 10–14% of women [3, 4, 11, 12]. SBRT as an alternative to BT boost has been investigated in small non-randomized studies with short follow-up periods, in these studies different EBRT techniques, volumes, and prescriptions were used [5, 9, 13].

Between 2004 and 2011, intensity-modulated radiation therapy (IMRT) and SBRT boost in the United States increased considerably, whereas the use of BT decreased by an estimated 10% [3]. However, IMRT and SBRT boosts have been linked to an increased risk of death (Hazard Ratio[HR]: 1.86, after controlling for significant factors affecting survival) [3]. A propensity-matched retrospective analysis of 15.905 CC patients was published, showing that IMRT (n = 1.468) significantly decreased OS compared to BT (n = 14.394), whereas SBRT boost (n = 42) had the same OS as BT (HR: 1.48, 95% IC 0.746–2.926; p = .263)) [4].

Published clinical outcomes following EBRT/SBRT boost are limited and provide contradictory data [14]. On multivariable analysis, it has been shown that older age, locally advance CC (IVA), tumor size, and treatment facilities were all correlated with lower BT boost usage [3]. Contradictorily, patients with advanced disease have a higher benefit from a BT boost, while they are also more likely to get an alternate treatment to BT [11]. As a result, neither SBRT nor IMRT are currently evidence-based substitutes for BT boost and should only be explored in the case of patients who are unsuitable due to medical contraindications [5, 9, 15].

To this day, SBRT boost is not recommended as standard treatment owing to the lack of well-powered comparative studies and its poorer OS compared to BT [1, 3, 7, 8].

In terms of BT, several techniques, models, and schedules are used, making it challenging to compare oncologic outcomes, treatment toxicity, and effective dosages [16]. By adding needles to the peripheral (and central) portions of the target volume, interstitial brachytherapy (IC + IS-BT) can improve BTs’ conformality, providing dose escalation while preserving OAR [8, 17]. IC + IS BT can be used to overcome difficult target coverage due to larger tumors, asymmetric tumors, OARs proximity, and patient anatomy [8, 17]. Indeed, the EMBRACE-I study investigation found that adding needles enhanced the target dosage, decreased OAR doses [17].

In CC, no data has been reported on the direct dosimetric comparison of IC-BT vs. IC + IS BT vs. SBRT, and they cannot be directly compared because of population heterogeneity. Indeed, each interstitial series is unique in terms of patient selection, size, and treatment approach [7]. Accordingly, the dosimetric differences between different boost modalities should be investigated by comparing them in the same patient rather than in different patient groups or historical controls, to have comparable volumes in terms of target and OAR. This study aims to compare dosimetric differences in terms of target coverage and OARs doses on an individual level between three distinct boost modalities in LACC: IC-BT alone, IC + IS-BT, and SBRT.

## Materials and methods

### Patients and design

24 consecutive treatment plans with histologically proven CC who have been treated with IGABT (IC + IS-BT) boost (4 × 7.1 Gy) after a Volumetric Arc Therapy (VMAT) (45-55 Gy/25) plus concomitant weekly cisplatin (40 mg/m^2^), were retrospectively investigated. The 24 treatment plans corresponded to 17 patients because 7 patients were treated twice with IC (tandem/ovoids) + IS-BT. The radiation oncologist implanted the intrauterine tube, ovoid, with 1 to 10 interstitial needles (Utrecht Interstitial applicator) under general anesthesia within the first week after CCRT. Pretreatment (clinical, radiological), surgical, and clinical data defined the optimal number of needles. The Jules Bordet Institut Ethics Committee allowed our investigation (1637752023498).

Following the IC + IS-BT application, a whole pelvis computed tomography scan (Aquilion™ Large Bore, 3-mm slice thickness), and a pelvic magnetic resonance imaging (MRI) simulation were performed as recommended by the GEC-ESTRO Working Group [18]. The GTV-T, HR-CTV, and OAR (bladder, rectum, sigmoid, and bowel bag) were contoured on the MRI, using the EMBRACE II protocol guidelines [6]. Volumes contoured on the MRI were transferred to the CT with co-registration performed by matching the applicator on the CT with the applicator on the MRI. The use of a Foley catheter with a clamp allowed for consistent bladder filling (100 cc) in patients with an empty rectum, reducing spatial variability. As no planning target volume (PTV) nor planning (organ at) risk volume (PRV) margins were used for any structure, all structures were identical for each boost modality.

### Treatment planning

All patients were treated with the microSelectron® Digital HDR (high dose rate). The Varian Medical System (Eclipse) was used to create a 3D IC + IS-BT plan. Dose-volume adaptation began with the activation of the uterine applicator and ovoid source positions, follow by a point normalization, then a manual dwell location and time optimization in the needles channels. All the treatment procedures described in this study were part of our department’s standard clinical practice. The equivalent doses in 2 Gy fractions (EQD_2_) of EBRT and BT for CTV-HR and OAR were calculated using the linear-quadratic model with α/β of 10 (EQD2_10_) and 3 (EQD2_3_), respectively. The EMBRACE II protocol was used during plan optimization [6].

For every IC + IS-BT treatment plan, one IC-BT plan and one SBRT plan were created. The plans were created using the same contouring volumes (target and OAR) as the IC + IS-BT plan and were calculated using the same CT scan. The uterine applicator and needle channels were converted to water density for the SBRT plans.

SBRT plans were created with a full-arc VMAT (360°) using 6MV photons based on the Monaco v5.0 treatment planning system (Elekta AB, Stockholm, Sweden). A 3 mm grid size was used for calculation, and 90 control points were allowed per arc. There were no maximum dose restrictions applied in the constraints nevertheless, the hotspots must lie within the HR-CTV.

Once the best possible treatment plans were created for the three modalities, two different normalizations were performed: (1) Normalization to the target: prescription of 7.1 Gy to the D90% (defined as the minimum dose covering 90%) of the HR-CTV, thereby enabling dose comparison between OARs of the different boost modalities; and (2) Normalization to the OAR to meet all the OAR’s hard constraints, thereby enabling comparison of HR-CTV dose coverage. Quantitative data are presented in median and interquartile ranges (Q1-Q3) or the standard deviation of the mean (SEM). The isodoses, between each plan were compared using the Paired Student T test, Repeated Measures ANOVA, or Wilcoxon signed-rank test, and the Friedman test. Statistical analysis was performed using R version 4.1.0, with a p-value < 0.05 considered significant.

## Results

The median (Q1-Q3) age was 55 years (44-63.5), BMI was 24.4 kg/m³ (21.75–31.61); HR-CTV was 27.48 (20.58–34.66) mm^3^ (Table 1). The majority of patients (71%) had a FIGO (2018) stage of ≥ IIB. The median number of IS needles implanted was 4 [4–6], and a median of 3 [2–4] were charged per patient. The total median needles contribution per fraction was 11.76% (10.34–19.83). The needle contribution was > 15% in 16 fractions, 20.67 (18-97-41.92). The median total air Kerma was 3442 (3020–3770) mGy, and the total treatment time was 470.7 (357.6-695.7) seconds. In the SBRT group, the Dmax were 10.71 (10.42–11.26) Gray (Table 1).

**Table 1:** Patients, tumor, and treatment characteristics

Variable	Value
Age (years) *Median (Q1-Q3)*	55 (44-63.5)
FIGO Stage (n)	
IB2	3
IIA	3
IIB	6
IIIA	1
IIIB	2
IIIC1	1
IIIC2	1
IVA	1
BMI (kg/m²) *Median (Q1-Q3)*	24.4 (21.75–31.61)
HR-CTV (cc) *Median (Q1-Q3)*	27.48 (20.58–34.66)
Needles	
*Median (Q1-Q3)*	
Needles implanted (n) Needles used during brachytherapy (+) Contribution (%)	4 (4–6) 3 (2–4) 11.76 (10.34–19.83)
Total air Kerma (mGy) *Median (Q1-Q3)*	3442 (3020–3770)
Total Treatment time (s)	470.7 (357.6-695.7)
SBRT (Dmax, Gy) *Median (Q1-Q3)*	10.71 (10.42–11.26)

*Abbreviation*: Q1-Q3: First quartile-third quartile; FIGO 2018: The International Federation of Gynecology and Obstetrics; BMI: Body Mass index; cc: cubic centimeter; HR-CTV: High-risk clinical target volume; mGy: milligray; SBRT: Stereotaxic Body Radiotherapy; Dmax: maximum dose; Gy: Gray

**Fig. 1:**
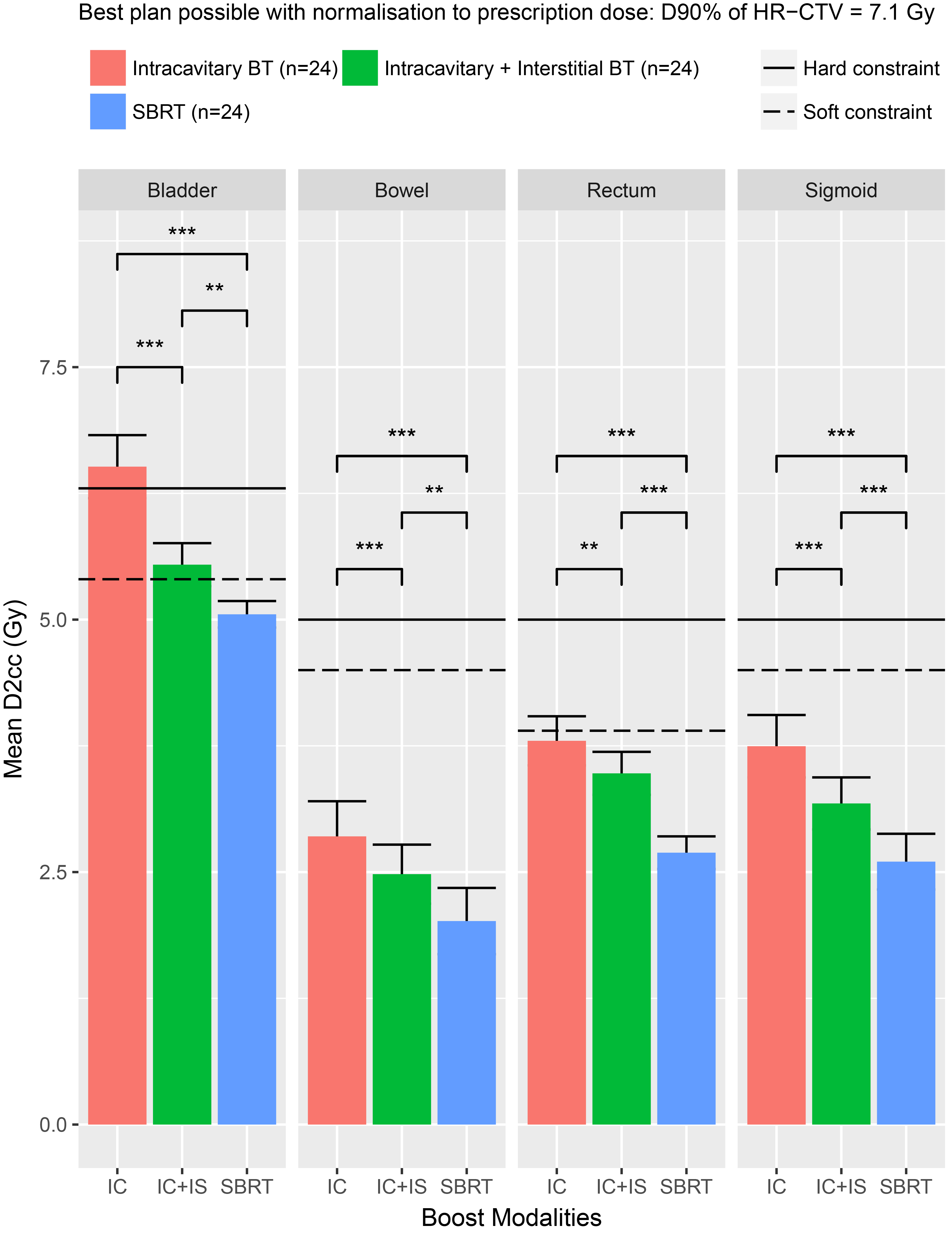
Dose to Organs at risk between intracavitary BT vs. intracavitary + interstitial BT vs. SBRT

When normalizing to the target, the mean EQD2_3__D2cc of OARs was significantly higher with IC-BT (Table 2). In particular, bladder EQD2_3_ D2cc hard constraints (< 90 Gy) were not achieved when boosting with IC-BT (Fig. 1). Adding IS needles to IC BT results in a median absolute reduction of bladder EQD2_3_ D2cc of 1 Gy (i.e. a relative dose reduction of 19%), thereby achieving the hard dose constraint (Fig. 1; Table 2). SBRT without PTV (vs. IC-BT and vs. IC + IS-BT) provides significantly lower OARs EQD2_3__D2cc dose to the bladder; bowel and rectum (Table 2). For the sigmoid constraints, there was no significant difference (p = .08) between the three boost modalities (Table 2).

**Table 2:** Dosimetric comparison between intracavitary BT vs. intracavitary + interstitial BT vs. SBRT

Structures	Mean (SEM) Dose (Gy)			IC-BT vs. SBRT	IC-BT vs. IC-BT + IS	IC-BT + IS vs. SBRT	IC-BT vs. IC-BT + IS vs. SBRT
	IC-BT	IC-BT + IS	SBRT				
**OAR’s normalization**							
D30_HR_CTV	14.9 (14.16-15-64)	15.2 (14.65–15.75)	10.1 (9.93–10.27)	P < .001	P = .37	P < .001	P < .001
D50_HR_CTV	11.1 (10.57–11.63)	12.1 (11.67–12.53)	9.83 (9.61–10.05)	P = .005	P < .001	P < .001	P = .001
D90_HR_CTV	6.62 (6.24-7)	7.8 (7.51–8.09)	9.02 (8.75–9.29)	P < .001	P < .001	P < .001	P < .001
**Target normalization**							
D2cc_Bladder	6.46 (6.13–6.79)	5.49 (5.25–5.73)	4.99 (4.85–5.13)	P < .001	P < .001	P = .002	P < .001
D2cc_Bowel	2.82 (2.47–3.17)	2.48 (2.16–2.8)	2.06 (1.69–2.43)	P < .001	P < .001	P = .07	P < .001
D2cc_Rectum	3.87 (3.6–4.14)	3.58 (3.34–3.82)	2.71 (2.54–2.88)	P < .001	P = .03	P < .001	P = .003
D2cc_Sigmoid	3.65 (3.3-4)	3.11 (2.8–3.41)	2.59 (2.27–2.91)	P < .001	P < .001	P < .001	P = .08

*Abbreviation*: SEM: standard deviation of mean; Gy: Gray; IC-BT: Intracavitary brachytherapy; IC-BT + IS: Intracavitary plus interstitial brachytherapy; SBRT: Stereotactic Body Radiotherapy; HR-CTV: High-risk clinical target volume; D30-50-90: defined as the minimum dose covering 30-50-90% of the volume; OARs: organs at risk; D2cc: defined as the minimal dose of the 2 cc the volume

**Fig. 2:**
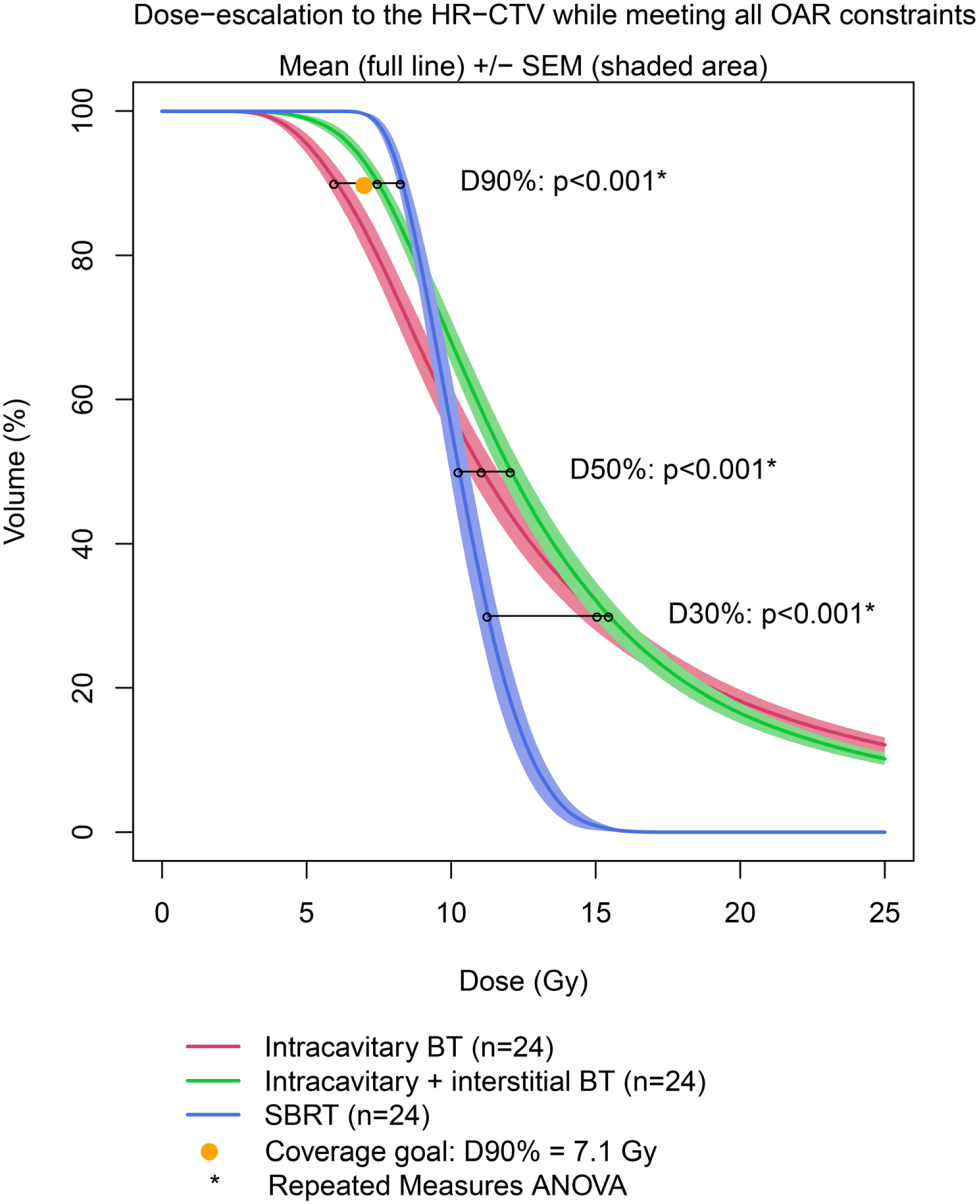
Mean DVH of High-Risk CTV (HR-CTV) between intracavitary BT vs. intracavitary + interstitial BT vs. SBRT

When normalizing to the OARs while escalating the dose to the target, IC-BT provides a significantly lower mean dose to the EQD2_10_ D90% of HR-CTV (6.62 Gy (6.24-7)) and cannot achieve the coverage goal of 7.1 Gy (Fig. 2; Table 2). SBRT without PTV delivers the highest dose to the D90% HR-CTV (9.02 Gy), but significantly lower doses to the D50% (9.83 Gy) and D30% (10.1 Gy) (Table 2). Between the IC-BT and IC-BT + IS modalities, the D30 HR CTV did not significantly change (p = .37) (Table 2).

## Discussion

Even though clinical guidelines suggest BT boost to be the gold standard in LACC, some centers still attempt to replace BT with an EBRT boost because of its practical advantages, despite the potential inferior clinical outcome [3, 4, 12, 14, 19]. In the present study, dosimetric differences in terms of target coverage and OARs doses were investigated between IC-BT, IC + IS-BT, and SBRT. To our knowledge, this is the first study reporting the dosimetric outcomes of different boost modalities using an identical dose prescription. The results of this study suggest the main benefit of BT over SBRT boost is the greater HR-CTV’s D50% and D30% (Fig. 2; Table 2). The ability to deliver a high local and conformal dose to the target while (better) sparing the OAR suggests BT to be (currently) indispensable for CC treatment [3, 4, 12].

SBRT allows radiation oncologists to deliver dose to the target in a very conformal manner over a few fractions, with a higher biologically equivalent dose than EBRT [14, 20]. At least seven recent trials have looked into SBRT boost in LACC patients unable to receive BT, five of which were retrospective [14, 16, 21–23] and two were prospective (phase I: [20] II: [24]). The majority of these studies have a short follow-up a limited number of patients and delivered different doses and fractionations. In addition, target volumes and planning techniques varied widely across studies. The median EQD2 dose equivalents to diverse CTV targets were between 73–104 Gy and 75–80 Gy in trials using SBRT and IMRT boost, respectively [9].

Depending on the baseline performance status, a wide variety of results have been reported on, including OS (46.9–100%) and progression-free survival (PFS) (25.9–100%) rates. Compared to other BT boost groups, SBRT patients are often older and have more comorbidities [9]. However, determining the therapeutic superiority of BT boost would demand not only dosimetric but also clinical and treatment quality assurance data.

Cengiz et al., compared SBRT (Cyberknife®) vs. IC (tandem + two Ovoids) HDR BT boost plans in 11 CC patients [13]. They found better tumor coverage with SBRT, for all reference isodose lines [13]. This might be due to the fact that they looked at a volume, larger than our HR-CTV, defined as “the whole cervix containing the tumor plus a 1 cm safety margin superiorly and inferiorly toward the vagina and the uterus” [13]. The use of a standard Point A prescription, as well as the use of CT rather than MR, were the main limitations for the BT group. In our cohort, SBRT yields the highest dose to the D90% of HR-CTV (9.02 Gy; p = .001); however, the D30% and D50% are in favor of the BT group (Table 2). Regarding the bladder and rectum, they also found a statistically significant lower D2cc in favor of the SBRT plans. It is essential to emphasize that our results were obtained without using any PTV or PRV margins, which is generally not recommended when treating with SBRT in a clinical setting.

Georg et al. compared an inverse boost plan from an MRI-guided BT method to external-beam photon and proton (PT) therapy in 9 LACC patients [25]. They computed a normalization to the OAR to maximize the dosage to the target, which is comparable to our work. However, they did not perform normalization to the target (OAR assessments). The GTV coverage was lower for both external procedures [25]. In PT plans, high-risk PTV (CTV + 3-mm margins) doses were equivalent to BT but inferior to photon plans [25]. Sharma et al. showed IC + IS-BT to be superior to IMRT (step-and-shoot) in 12 patients who were not candidates for brachytherapy [26]. When compared to IMRT, BT outperformed in terms of EQD2_10_ D_95_ PTV coverage (57.16 vs. 41.47 Gy; p = .003) and Bladder EQD2_3_ D1cc (50.64 vs. 66.31 Gy; p = .004) [26]. When comparing IC + IS-BT to IMRT, BT improved the rectal EQD2_3_ D1cc, which might be related to the limitations of step-and-shoot IMRT procedures and the PTV margins (3-5 mm) used around the IC + IS-BT target [26]. However, they did not apply MR-based target delineation like Cengiz et al. did [13].

Studies comparing EBRT plans to BT have been criticized for employing insufficient PTV margins, especially in the EBRT arms [13]. Internal motion between and within fractions represents a significant obstacle when using EBRT as a substitute for BT Boost, possibly contributing to local failures and/or complications. The PTV defined by Georg et al. was 3 mm “optimistic” margins, and the OARs had no PRV [25], although it is known that the cervix can move up to 18 mm from its initial position, with an average of 3 mm in either direction [27]. In our study, no PTV or PRV margins were applied for any structure, hence, all structures were identical for each boost method. The goal of not using a PTV margin for SBRT was to evaluate the pure dosimetric differences between the different modalities using the exact same volumes for target and OAR. Of course, in clinical practice, we would recommend applying a PTV margin when using EBRT or SBRT, and the size of the PTV margin should be selected based on the technique and the image guidance used. If a PTV margin was added to the SBRT plans, the dose to the OAR would inevitably be higher and this should be carefully considered when evaluating our results.

According to our findings, the key benefit of BT over SBRT is the greater D50% and D30% to the HR-CTV. The EMBRACE I study found 98% of local failures to occur inside the CTV-HR (and CTV-IR), hence validating the GEC-ESTRO response-adapted target volume concept [6]. The HR-CTV D90 > 86–92 Gy (total dose, EQD2_10_) resulted in a LC > 90–95% with a robust dose-effect association [6, 7]. In our study, IC-BT provided a significantly lower dosage to the D90% of the HR-CTV as compared to IC + IS-BT and SBRT (Fig. 2; Table 2). By adding needles to the peripheral (and central) portions of the target volume, IC + IS-BT can improve BT’s conformality, providing dose escalation while sparing the OARs [8, 17].

Although D2cc has been proven to correspond with toxicities of the bladder and rectum, no consistent link was found for the vaginal mucosa, sigmoid, urethra, or ureters [7].

Rectal dosimetry suggests an EQD2_3__D2cc < 60–75 Gy. The rectum dose difference between IC-BT and IC + IS-BT was small (3.87 vs. 3.58 Gy; p = .03), especially as compared to the bladder (6.46 vs. 5.49 Gy; p < .001) (Fig. 1; Table 2).

The EQD2_3__D2cc of the sigmoid, was not different (p = .08) between the three boost modalities. This might be explained by the fact that the sigmoid structure is rarely near to the HR-CTV.

Compared to SBRT, IC + IS-BT and IC achieve a significantly higher Mean EQD2_3__D2cc exposure of the OARs (Fig. 1; Table 2). However, since no PTV margin was used for the SBRT plans, this difference can be undone in a comparison between BT without PTV and SBRT with PTV. IC delivers substantially greater dose to the OAR than SBRT and IC + IS-BT, because of the high doses administered straight from one single IC BT device located close to the OARs.

Some of the difficulties in decreasing the incidence of complications come from the lack of knowledge about the “optimal” technique to improve boost dosimetry. In the boost setting, the idea that EBRT (IMRT/SBRT) can produce a homogenous dosage is irrelevant, and the clinical consequences are unknown [9, 25]. Typically, the aim of the boost is to focus on the dose (para)-centrally, where hypoxic cells abound and recurrences are the most prevalent [9]. High-dose zones in the center, close to BT sources, can reach > 200–300% of the prescribed dose [9, 25]. The superior clinical outcome of BT in retrospective studies might be explained by its heterogeneity, which is defined as hot and cold regions with fewer low-dose surroundings, resulting in the capacity to preserve certain immune island cells. However, future studies are needed to explore this hypothesis. Spatially Fractionated Radiation Therapy (SFRT) techniques have recently demonstrated promising outcomes in various bulky tumors [28]. The extreme heterogeneity of SFRT mimics the dose distribution of BT, and especially the dose distribution of IC + IS-BT.

Our study has some limitations, the main being that this is a dosimetric in silico study. As the IC-BT and SBRT plans were created retrospectively for research purposes only, we do not have clinical outcome data on them. Especially the choice not to add a PTV margin for the SBRT plans in order to compare the pure dosimetric differences makes it questionable whether the SBRT plans are clinically deliverable and provide a dosimetric advantage for the SBRT plans.

## Conclusion

The main advantage of BT in comparison to SBRT is the higher D50% and D30% to the HR-CTV. Dose escalation of BT naturally occurs at the center of the target and might explain the superior outcome of BT in epidemiological series. IC + IS-BT provides a significantly better target coverage and lower dose to the OARs than IC-BT, and therefore seems dosimetrically the best boost modality in CC.

## Data Availability

Not applicable.

## Abbreviations

CC: Cervical cancer
IC: Intracavitary
BT: Brachytherapy
IS: Interstitial
SBRT: Stereotactic-Body-Radiotherapy
OAR: Organ at risk (OAR)
HR-CTV: High-risk clinical target volume
EBRT: External beam radiotherapy
CCRT: Concomitant chemoradiotherapy
BT: Brachytherapy
LACC: Locally advanced cervical cancers
LC: Local control
OS: Overall survival
IGABT: Image-guided adaptive BT
IMRT: Intensity-modulated radiation therapy
VMAT: Volumetric Arc Therapy
PTV: Planning target volume
PRV: Planning (organ at) risk volume
HDR: High dose rate
PFS: Progression-free survival
PT: Proton
SFRT: Spatially Fractionated Radiation Therapy
D90%: Minimum dose covering 90% of the volume
D2CC: Minimal dose of the 2 cc of the volume
